# Risk factors for postoperative complications after UBE surgery for thoracic spinal stenosis and construction of a nomogram predictive model

**DOI:** 10.3389/fneur.2025.1616590

**Published:** 2025-08-21

**Authors:** Mingkui Shen, Lulu Wang, Zhongxin Tang, Xiaohu Wang, Hejun Yang

**Affiliations:** ^1^Department of Mini-invasive Spinal Surgery, The Third People’s Hospital of Henan Province, Zhengzhou, Henan, China; ^2^Henan Engineering Research Center of Precision Diagnosis and Treatment of Intervertebral Disc Disease, Zhengzhou, Henan, China; ^3^Department of Plastic Surgery, The Third People’s Hospital of Henan Province, Zhengzhou, Henan, China; ^4^Department of Orthopedics, Zhengzhou Central Hospital, Zhengzhou, China

**Keywords:** risk factors, thoracic spinal stenosis, unilateral biportal endoscopy, complication, nomogram model

## Abstract

**Background:**

This study aimed to develop and validate the first nomogram model for predicting postoperative complications in thoracic spinal stenosis (TSS) patients undergoing unilateral biportal endoscopy (UBE), integrating multidimensional risk factors to provide a quantitative basis for preoperative risk evaluation and individualized treatment planning.

**Methods:**

Patients were divided into a retrospective training cohort (*n* = 375) and a prospective validation cohort (*n* = 100). Baseline clinical data [age, diabetes, preoperative Japanese Orthopaedic Association (JOA) score], radiographic parameters (Spinal cord/canal area (SC/ECA) ratio, intramedullary high signal, thoracic kyphosis (TK) angle), and surgical variables (intraoperative blood loss, number of lesion segments, dural adhesion, etc.) were collected. Independent risk factors were identified using logistic regression analysis, and a nomogram model was constructed. Model performance was assessed using receiver operating characteristic (ROC) curves, calibration curves, and decision curve analysis (DCA).

**Results:**

In the training cohort, 30 patients experienced postoperative complications (37 total events), while 10 patients in the validation cohort had complications (19 total events). Major complications included cerebrospinal fluid leakage, neurological deterioration, poor wound healing, and epidural hematoma. Multivariate logistic regression analysis revealed that diabetes, SC/ECA ≥ 55%, intramedullary high signal, TK angle ≥ 45 °, dural adhesion, multisegment lesion, increased intraoperative blood loss, and prolonged hospitalization were independent risk factors, whereas a higher preoperative JOA score was protective. The nomogram demonstrated excellent discrimination (AUC = 0.964 for training cohort; 0.846 for validation cohort) and good calibration in both cohorts. DCA indicated significant clinical net benefit when the threshold probability exceeded 10%, especially for identifying high-risk patients (threshold > 40%). Risk weight analysis showed that multisegment lesion (25 points) and SC/ECA ≥ 55% (20 points) contributed most to complication risk, followed by intramedullary high signal (15 points) and TK angle (15 points).

**Conclusion:**

This study successfully established a predictive nomogram for postoperative complications following UBE in TSS patients. The model demonstrated high accuracy and clinical utility, providing valuable guidance for preoperative risk stratification and perioperative management, thereby promoting precision in minimally invasive thoracic spine surgery.

## Introduction

Thoracic spinal stenosis (TSS) is one of the most challenging conditions in the field of spinal surgery. With the global trend of population aging, the incidence of TSS is increasing annually. For instance, Korea scholars Moon et al. (1) evaluated MRI findings in 2,134 patients (with back pain or low back pain) and reported an ossification of ligamentum flavum (OLF) prevalence of 16.9% (OLF being the most common cause of TSS), which increased with age. Similarly, Japanese scholars Mori et al. (2) analyzed CT scans of 3,010 patients (screened for pulmonary diseases) and found an OLF prevalence of 36%. In a comparable study, Chinese researchers Lang et al. (3) standardized prevalence rates according to age demographics, revealing a remarkably high adjusted OLF prevalence of 63.9%. This progressive rise has established TSS as a major health issue affecting the quality of life in middle-aged and elderly patients. Notably, clinical studies have shown that approximately 52.1% of TSS patients also present with degenerative changes at other spinal levels, making diagnosis and treatment significantly more complex (4). Although traditional open surgery can effectively decompress the spinal cord, its large surgical trauma and high complication rate limit its clinical application. Several studies have reported complication rates of up to 25% following conventional posterior laminectomy and decompression procedures (5, 6). Recently, the rapid development of minimally invasive spinal techniques has introduced unilateral biportal endoscopy (UBE) as a promising alternative for TSS treatment. Compared with traditional open surgery, UBE offers significant minimally invasive advantages, including better visualization of the surgical field and reduced paraspinal muscle dissection (7). Clinical research indicates that patients undergoing UBE experience an average reduction in intraoperative blood loss by 62%, a 3.5-day shorter postoperative hospital stays, and faster early functional recovery (8, 9). Particularly in elderly patients or those with multiple comorbidities, UBE provides a safer surgical option, with the 30-day postoperative complication rate being approximately 40% lower than that of open procedures (10).

However, despite the overall lower complication rate of UBE compared to open surgery, specific complications such as epidural hematoma and cerebrospinal fluid (CSF) leakage remain non-negligible (11). More importantly, most existing predictive models are based on data from open surgeries and are not fully applicable to the UBE technique. For example, UBE’s restricted working corridor increases the risk of dural tears and neural injury due to instrument crowding. Meanwhile, its fluid-mediated visualization system may predispose to unique complications like epidural hematoma from sustained irrigation pressure. As a result, risk assessment in current UBE procedures largely depends on the surgeon’s experience, with a lack of systematic predictive tools. A multicenter survey revealed that a majority of spinal surgeons believe that current risk assessment methods are inadequate for UBE surgery (12). This gap between clinical demand and technological capability highlights the importance and urgency of developing UBE-specific complication prediction models.

This study aims to innovatively construct the first nomogram model for predicting postoperative complications in TSS patients undergoing UBE. Through systematic integration of multidimensional clinical data encompassing baseline characteristics, radiographic parameters, and intraoperative variables, the model offers a comprehensive visualization of each risk factor’s predictive contribution. This quantitative tool enables precise preoperative risk stratification, helping surgeons to identify high-risk patients and optimize perioperative management strategies. Ultimately, the proposed model has the potential to significantly reduce the incidence of complications following UBE and to fill the current gap in risk assessment within the context of minimally invasive spinal surgery. Moreover, it offers valuable support for the standardized and precise development of UBE techniques in clinical practice.

## Methods

### Study population

This study was conducted in two phases. In the first phase, a retrospective analysis was performed on clinical data from patients who underwent UBE surgery for TSS at The Third People’s Hospital of Henan Province between March 2020 and March 2023. Eligible patients were included in the model development cohort for predicting postoperative complications following UBE surgery. Inclusion criteria: a. Age between 18 and 75 years; b. Diagnosed with TSS based on clinical and radiological findings; c. Treated with UBE surgery; d. Complete follow-up data for ≥ 12 months, including postoperative complications and laboratory results. Exclusion criteria: a. Severe cardiac, hepatic, or renal dysfunction; b. Preoperative neurological dysfunction (ASIA grade ≤ C); c. Coexisting spinal disorders (cervical spondylosis, lumbar disc herniation); d. Intraoperative conversion to open surgery or modification of surgical procedure. A total of 375 patients were ultimately included in the training cohort.

In the second phase, a prospective observational study was conducted to validate the predictive model. Patients with TSS treated between March 2023 and March 2024 at The Third People’s Hospital of Henan Province were enrolled. Inclusion criteria: Same as criteria a – c for the training cohort, and patients provided informed consent after being fully informed about the study. Exclusion criteria: a. History of spinal trauma, surgery, or related treatments; b. Severe comorbidities affecting surgery or prognosis (cardiac, hepatic, renal, or immune disorders); c. Preexisting neurological dysfunction; d. Coexisting spinal diseases (cervical spondylosis, lumbar disc herniation); e. Severe cognitive impairment or psychiatric disorders; f. Inability or unwillingness to complete assessments; g. Pregnancy or lactation; h. Participation in other clinical trials within the past 3 months.

A total of 100 patients were enrolled as the validation cohort. Patients in both cohorts were divided into complication present (CP) and complication absent (CN) groups based on the occurrence of postoperative complications.

### Clinical data collection

#### Baseline data

Collected variables included age, sex, body mass index (BMI), disease duration, comorbidities (diabetes, cardiovascular disease, hypertension), and preoperative Japanese Orthopaedic Association (JOA) scores. The JOA score (9-point scale) assessed neurological function based on lower limb motor function, lower limb and trunk sensation, and bladder function. Higher scores indicate better neurological status.

#### Imaging parameters

Spinal cord/canal area ratio: On high-resolution T2-weighted axial MRI, the most stenotic segment was selected. The spinal cord cross-sectional area (SCA, excluding CSF) and the effective canal area (ECA, traced along the bony boundary) were manually measured using Image J. The ratio was calculated as (SCA/ECA) × 100%. A ratio ≥ 55% was considered severe stenosis. The measurement was repeated three times and averaged.

T2-weighted high signal: Defined as the presence of a focal or diffuse hyperintensity on T2WI at or near the lesion level. All images were independently evaluated by two neuroradiologists (10 + years’ experience), with discordant cases resolved through consensus review.

Etiology of stenosis: Based on CT and MRI findings, compressive lesions were classified as either soft (herniated discs) or hard (ossified posterior longitudinal ligament, ossification of ligamentum flavum, calcified discs, osteophytes).

Thoracic kyphosis (TK) angle: Measured on lateral X-rays of the entire thoracic spine using the Cobb method; angles > 45 ° were considered abnormal.

#### Surgical data

Surgical data included operative time, estimated intraoperative blood loss, number of lesion segments, intraoperative use of methylprednisolone, and postoperative hospital stays.

### Definition of postoperative complications

Patients were followed up at 1, 3, 6, and 12 months postoperatively. The composite endpoint for complications included both direct and indirect adverse outcomes occurring within 12 months after surgery. For the validation cohort, the follow-up period extended until March 2025.

#### Direct complications

CSF leakage, epidural hematoma, spinal cord or nerve root injury, transient neurological deterioration, surgical site infection, and fat liquefaction were observed.

#### Indirect complications

Adverse outcomes including mortality, pneumonia, deep venous thrombosis (DVT), pressure ulcers, and urinary tract infections were assessed.

#### Assessment criteria for major complications

CSF leakage: a. Intraoperative dural tear; b. Postoperative drainage > 300 mL/day; c. Positive *β*-2 transferrin test in drainage fluid; d. Classic postural headache symptoms.

Epidural hematoma: a. Neurological deterioration within 24 h post-operation; b. Emergency MRI showing epidural mass effect.

Neurological injury: a. Significant motor/sensory loss; b. Intraoperative neurophysiological monitoring abnormalities; c. Abnormal spinal cord/nerve root signals on MRI; d. No recovery within 72 h.

Transient neurological symptoms: a. Temporary neurological decline within 72 h post-operation; b. Mild symptoms with spontaneous recovery.

All assessments were conducted in conjunction with preoperative baseline status using standardized tools (JOA scores), with dynamic evaluation at 6 h, 24 h, and 72 h post-operation. MRI was the preferred imaging modality; CT was used when necessary.

### Statistical analysis

Data were analyzed and graphed using SPSS 25.0 and GraphPad Prism. Categorical variables were expressed as n (%) and compared using the chi-square (*χ*^2^) test. Continuous variables were presented as ( $\bar{x}$ ± s) and compared using independent-samples t-tests. Logistic regression was used to identify risk factors associated with postoperative complications following UBE in TSS patients. A nomogram prediction model was developed using R software (version 4.4.1) and the “rms” package. To assess the model’s performance and clinical utility, ROC curves, calibration plots, and decision curve analysis (DCA) were employed. External validation was conducted using the prospective validation cohort to evaluate the model’s generalizability and predictive accuracy.

## Results

### Summary of postoperative complications

**Figure 1:**
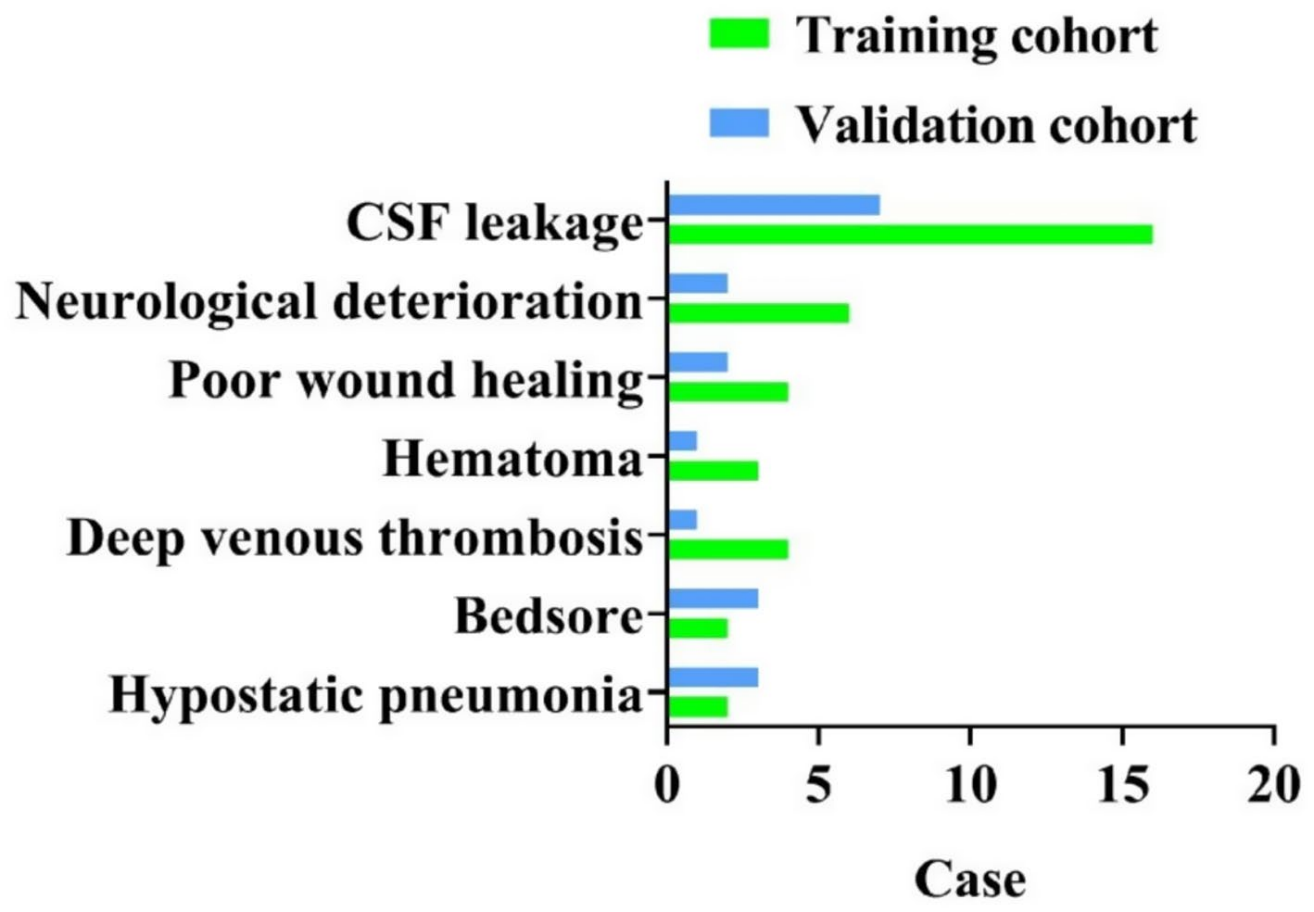
Distribution of postoperative complications.

In the training cohort, 30 patients experienced a total of 37 postoperative complications; in the validation cohort, 10 patients developed 19 complications. As some patients presented with multiple complications, the total number of events exceeded the number of affected patients. The main complications included CSF leakage, postoperative neurological deterioration, poor wound healing, DVT, epidural hematoma, bedsore, and hypostatic pneumonia (Table 1; Figure 1).

**Table 1:** Incidence of postoperative complications [*n* (%)].

Variable	Patients with complications in training cohort (*n* = 30)	Patients with complications in validation cohort (*n* = 10)
Cerebrospinal fluid leakage	16	7
Neurological deterioration	6	2
Poor wound healing	4	2
Hematoma	3	1
Deep venous thrombosis	4	1
Bedsore	2	3
Hypostatic pneumonia	2	3

### Comparison of baseline characteristics between training and validation cohorts

In the training cohort, there were no significant differences between the CP and CN groups regarding age, sex, BMI, disease duration, hypertension, or cardiac disease, suggesting comparability in demographic and comorbidity profiles. However, the CP group had a higher prevalence of diabetes and markedly lower preoperative JOA scores, indicating that diabetes and poor preoperative neurological function may be risk factors for postoperative complications (Table 2). Similarly, in the validation cohort, the CP group exhibited a higher prevalence of diabetes and lower preoperative JOA scores, with other baseline characteristics showing no significant differences (Table 3).

**Table 2:** Baseline characteristics in the training cohort [*n* (%), ( $\bar{x}$ ± s)].

Variable	CP group (*n* = 30)	CN group (*n* = 345)	t/χ^2^	*p*
Age (years)				
≥ 60	21 (70.00)	223 (64.64)	0.349	0.554
< 60	9 (30.00)	122 (35.36)		
Sex (cases)				
Male	17 (56.67)	186 (53.91)	0.084	0.772
Female	13 (43.33)	159 (46.09)		
BMI (Kg/m^2^)	25.42 ± 2.13	24.89 ± 2.59	1.089	0.277
Course of disease (months)	19.26 ± 14.32	23.35 ± 19.34	−1.131	0.259
Diabetes				
Yes	19 (63.33)	115 (33.33)	10.817	0.001
No	11 (36.67)	230 (66.67)		
Hypertension				
Yes	16 (53.33)	205 (59.42)	0.423	0.516
No	14 (46.67)	140 (40.58)		
Heart disease				
Yes	6 (20.00)	84 (24.35)	0.286	0.593
No	24 (80.00)	261 (75.65)		
Preoperative JOA score	4.65 ± 1.14	5.68 ± 1.28	−4.262	<0.001

**Table 3:** Baseline characteristics in the validation cohort [*n* (%), ( $\bar{x}$ ± s)].

Variable	CP group (*n* = 10)	CN group (*n* = 90)	t/χ^2^	*p*
Age (years)				
≥60	6 (60.00)	64 (71.11)	0.529	0.467
<60	4 (40.00)	26 (28.89)		
Sex (cases)				
Male	7 (70.00)	53 (58.89)	0.463	0.496
Female	3 (30.00)	37 (41.11)		
BMI (Kg/m^2^)	24.84 ± 2.25	25.69 ± 2.37	−1.081	0.282
Course of disease (months)	21.31 ± 14.68	22.98 ± 19.53	−0.262	0.794
Diabetes				
Yes	8 (80.00)	37 (41.11)	5.499	0.019
No	2 (20.00)	53 (58.89)		
Hypertension				
Yes	6 (60.00)	52 (57.78)	0.018	0.893
No	4 (40.00)	38 (42.22)		
Heart disease				
Yes	4 (40.00)	27 (30.00)	0.421	0.517
No	6 (60.00)	63 (70.00)		
Preoperative JOA score	4.12 ± 1.09	5.87 ± 1.31	−4.065	< 0.001

### Comparison of imaging characteristics

In the training cohort, the etiology of TSS did not markedly differ between groups. However, the CP group showed markedly higher rates of SC/ECA ≥ 55%, presence of intramedullary high signal intensity, and TK angle ≥ 45 ° (Table 4). The validation cohort showed similar findings, with SC/ECA ≥ 55%, positive intramedullary high signal, and TK angle ≥ 45 ° more prevalent in the CP group, while other imaging features were comparable (Table 5).

**Table 4:** Imaging characteristics in the training cohort [*n* (%), ( $\bar{x}$ *± s)].*

Variable	CP group (*n* = 30)	CN group (*n* = 345)	t/χ^2^	*p*
Etiology of spinal stenosis				
Ossified posterior longitudinal ligament	5 (16.67)	47 (13.62)	0.911	0.923
Ossification of ligamentum flavum	11 (36.67)	111 (32.17)		
Calcified discs	1 (3.33)	17 (4.93)		
Osteophytes	0	3 (0.87)		
Herniated discs	13 (43.33)	167 (48.41)		
SC/ECA ratio				
≥ 55%	23 (76.67)	172 (49.86)	7.949	0.005
40–55%	7 (23.33)	173 (50.14)		
Intramedullary high signal				
Yes	13 (43.33)	67 (19.42)	9.404	0.002
No	17 (56.67)	278 (80.58)		
Thoracic kyphosis				
≥ 45 °	21 (70.00)	145 (42.03)	8.753	0.003
< 45 °	9 (30.00)	200 (57.97)		

**Table 5:** Imaging characteristics in the validation cohort [*n* (%), ( $\bar{x}$ *± s)].*

Variable	CP group (*n* = 10)	CN group (*n* = 90)	t/χ^2^	*p*
Etiology of spinal stenosis				
Ossified posterior longitudinal ligament	2 (20.00)	10 (11.11)	3.232	0.520
Ossification of ligamentum flavum	5 (50.00)	28 (31.11)		
Calcified discs	0	7 (7.78)		
Osteophytes	0	3 (3.33)		
Herniated discs	3 (30.00)	42 (46.67)		
SC/ECA ratio				
≥ 55%	8 (80.00)	40 (44.44)	4.558	0.033
40–55%	2 (20.00)	50 (55.56)		
Intramedullary high signal				
Yes	6 (60.00)	23 (25.56)	5.186	0.023
No	4 (40.00)	67 (74.44)		
Thoracic kyphosis				
≥ 45 °	7 (70.00)	32 (35.56)	4.488	0.034
< 45 °	3 (30.00)	58 (64.44)		

### Comparison of surgical data

In the training cohort, there were no significant differences in operative time, decompression and fusion procedures, or postoperative drainage. However, the CP group had markedly higher intraoperative blood loss, a greater proportion of multi-segmental lesions, higher incidence of intradural adhesions, and longer hospital stays. Additionally, the use of intraoperative methylprednisolone was markedly lower in CP group (Table 6). Similar trends were observed in the validation cohort, with higher blood loss, multi-segment involvement, intradural adhesions, and longer hospitalization in the CP group; other variables were comparable (Table 7).

**Table 6:** Surgical data in the training cohort [*n* (%), ( $\bar{x}$ *± s)].*

Variable	C group (*n* = 30)	NC group (*n* = 345)	t/χ^2^	*p*
Surgical time (min)	185.25 ± 86.23	169.23 ± 71.68	1.154	0.249
Intraoperative bleeding volume (mL)	216.25 ± 64.36	152.23 ± 45.73	7.089	< 0.001
Number of lesion segments				
Multi segment	7 (23.33)	42 (12.17)	9.585	0.008
Double segment	20 (66.67)	204 (59.13)		
Single segment	3 (10.00)	99 (28.70)		
Intravertebral herniation with dural adhesion				
Yes	13 (43.33)	75 (21.74)	7.166	0.007
No	17 (56.67)	270 (78.26)		
Decompression fusion				
Yes	8 (26.67)	86 (24.93)	0.044	0.833
No	22 (73.33)	259 (75.07)		
Postoperative drainage				
Obstructed	17 (56.67)	170 (49.28)	0.603	0.437
Unobstructed	13 (43.33)	175 (50.72)		
Intraoperative use of methylprednisolone				
No	24 (80.00)	191 (55.36)	6.849	0.009
Yes	6 (20.00)	154 (44.64)		
Hospital stays (d)	12.5 ± 3.28	10.23 ± 2.68	4.366	< 0.001

**Table 7:** Surgical data in the validation cohort [*n* (%), ( $\bar{x}$ *± s)].*

Variable	C group (*n* = 10)	NC group (*n* = 90)	t/χ^2^	*p*
Surgical time (min)	202.25 ± 96.36	179.84 ± 78.57	0.837	0.405
Intraoperative bleeding volume (mL)	248.76 ± 69.45	161.38 ± 47.89	5.216	< 0.001
Number of lesion segments				
Multi segment	4 (40.00)	9 (10.00)	7.669	0.022
Double segment	5 (50.00)	54 (60.00)		
Single segment	1 (10.00)	27 (30.00)		
Intravertebral herniation with dural adhesion				
Yes	6 (60.00)	24 (26.67)	4.762	0.029
No	4 (40.00)	66 (73.33)		
Decompression fusion				
Yes	3 (30.00)	32 (35.56)	0.122	0.727
No	7 (70.00)	58 (64.44)		
Postoperative drainage				
Obstructed	6 (60.00)	35 (38.89)	1.658	0.198
Unobstructed	4 (40.00)	55 (61.11)		
Intraoperative use of methylprednisolone				
No	8 (80.00)	51 (56.67)	2.026	0.155
Yes	2 (20.00)	39 (43.33)		
Hospital stays (d)	13.2 ± 3.35	10.12 ± 2.58	3.474	< 0.001

### Risk factors of complications after UBE for TSS and their assignment

Variables with significant differences in univariate analysis from the training cohort, including diabetes, preoperative JOA score, SC/ECA ratio, intramedullary high signal, TK angle, intraoperative blood loss, number of lesion segments, intradural adhesion, intraoperative methylprednisolone use, and length of hospital stays, were included in the logistic regression analysis (Table 8).

**Table 8:** Risk factors and their variable assignments for complication.

Factor	Variable	Assignment
Diabetes	X1	No, 0; Yes, 1
Preoperative JOA score	X2	Actual measurement value
SC/ECA	X3	<55%, 0; ≥55%, 1
Intramedullary high signal	X4	No, 0; Yes, 1
Thoracic kyphosis	X5	<45 °, 0; ≥45 °, 1
Intraoperative bleeding volume	X6	Actual measurement value
Number of lesion segments	X7	Single segment, 1; Double segment, 2; Multisegment, 3
Intravertebral herniation with dural adhesion	X8	No, 0; Yes, 1
Intraoperative use of methylprednisolone	X9	Yes, 0; No, 1
Hospital stays (d)	X10	Actual measurement value

### Multivariate logistic regression analysis of risk factors for postoperative complications after UBE

Multivariate logistic regression analysis using SPSS 25.0 identified diabetes, SC/ECA ≥ 55%, intramedullary high signal, TK angle ≥ 45 °, intradural adhesion, increased intraoperative blood loss, multi-segmental lesions, and prolonged hospital stay as independent risk factors for postoperative complications after UBE. A higher preoperative JOA score was identified as a protective factor (Table 9). Notably, in analyzing the lesion segments as a risk factor, we combined single- and double segment cases as the reference group.

**Table 9:** Multivariate logistic regression of risk factors for postoperative complications after UBE.

Factor	B	SE	Wald	*p*	OR	95% CI
Diabetes	1.393	0.501	7.718	0.005	4.026	1.507–10.757
Preoperative JOA score	−0.83	0.192	18.647	0.000	0.436	0.299–0.636
SC/ECA	1.127	0.529	4.53	0.033	3.086	1.093–8.711
Intramedullary high signal	1.163	0.53	4.814	0.028	3.199	1.132–9.039
Thoracic kyphosis	1.092	0.502	4.728	0.03	2.98	1.114–7.976
Intraoperative bleeding volume	0.037	0.006	35.151	0.000	1.038	1.025–1.051
Number of lesion segments			9.092	0.011		
Double segment	1.874	0.76	6.081	0.014	6.512	1.469–28.873
Multisegment	2.676	0.898	8.885	0.003	14.524	2.5–84.371
Intravertebral herniation with dural adhesion	1.106	0.499	4.923	0.026	3.023	1.138–8.032
Hospital stays (d)	0.252	0.091	7.651	0.006	1.287	1.076–1.539
Intraoperative use of methylprednisolone	0.637	0.546	1.36	0.244	1.891	0.648–5.519

### Development and validation of the nomogram prediction model

#### Nomogram construction

**Figure 2:**
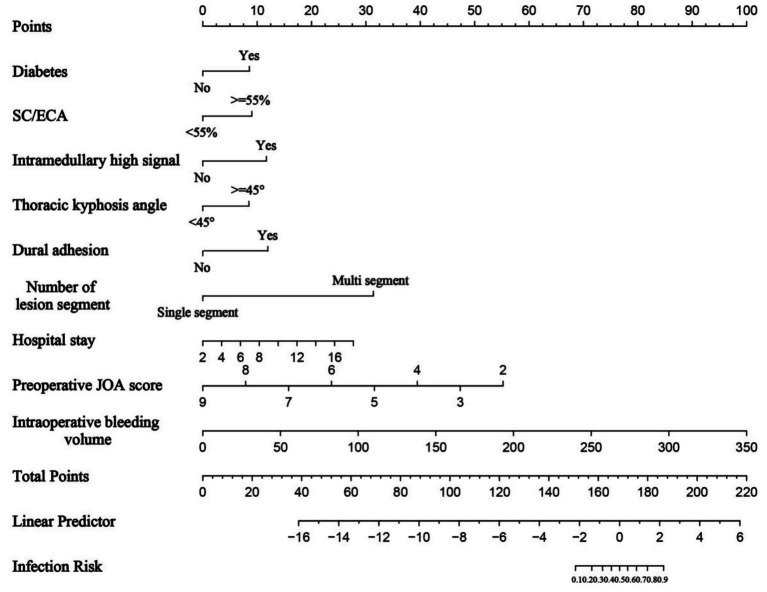
Nomogram for predicting postoperative complications. The higher the total score, the greater the predicted risk.

When constructing the nomogram model, the number of lesion segments was treated with dummy variable: single segment = 0; double and multiple segments = 1. The nomogram model indicated that the highest weighted factor for predicting postoperative complications was multi-segmental lesions (25 points), followed by SC/ECA ≥ 55% (20 points), intramedullary high signal (15 points), TK angle ≥ 45 ° (15 points), diabetes (10 points), and intradural adhesion (10 points). Lower preoperative JOA scores, greater intraoperative blood loss, and longer hospitalization were associated with higher total points (Figure 2).

#### Nomogram validation

**Figure 3:**
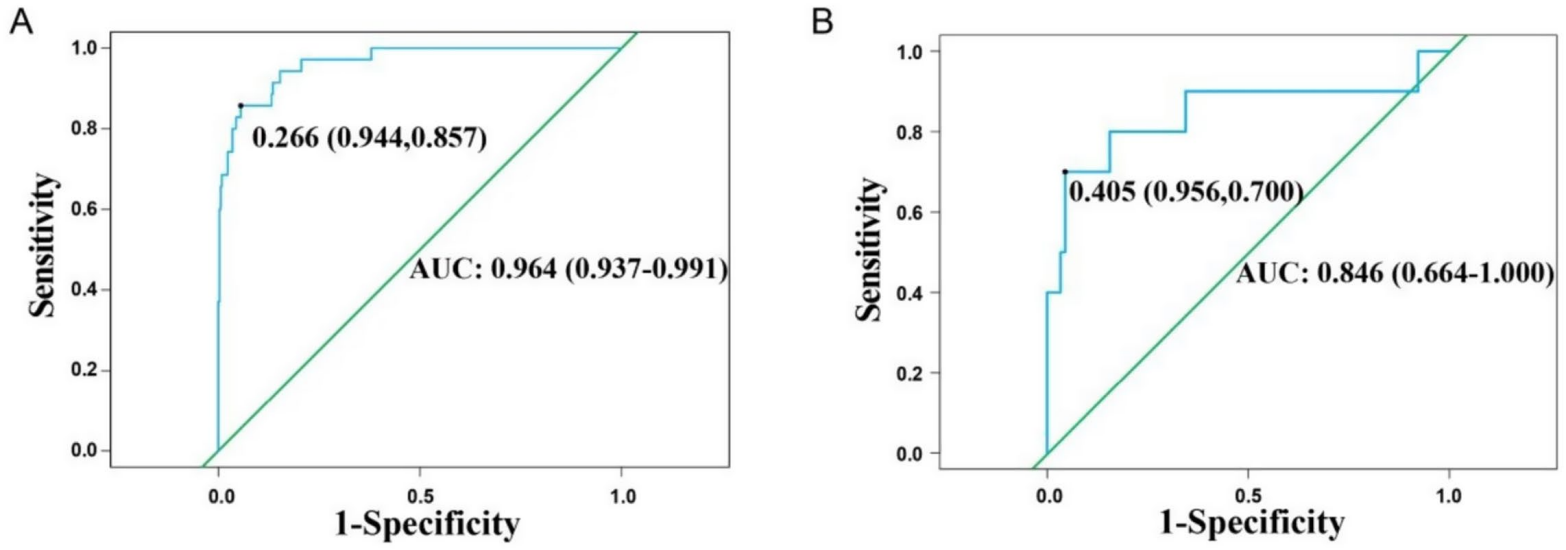
Receiver operating characteristic curves for training group **(A)** and validation group **(B)**.

**Figure 4:**
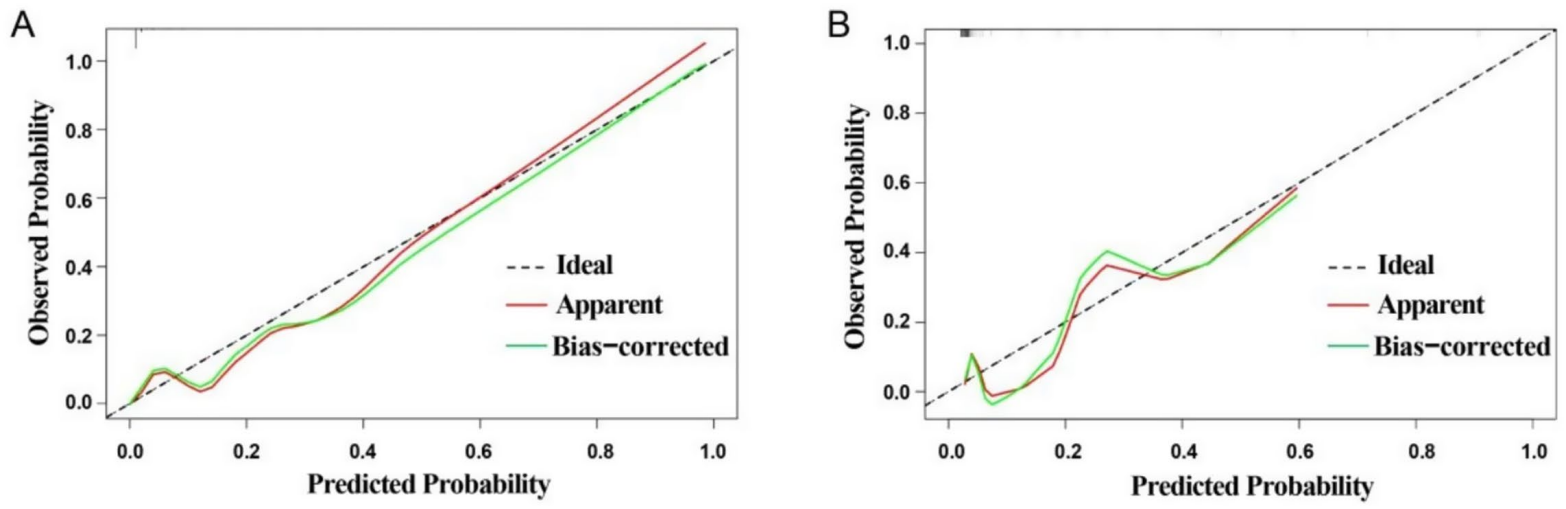
Calibration curves for training group **(A)** and validation group **(B)**.

**Figure 5:**
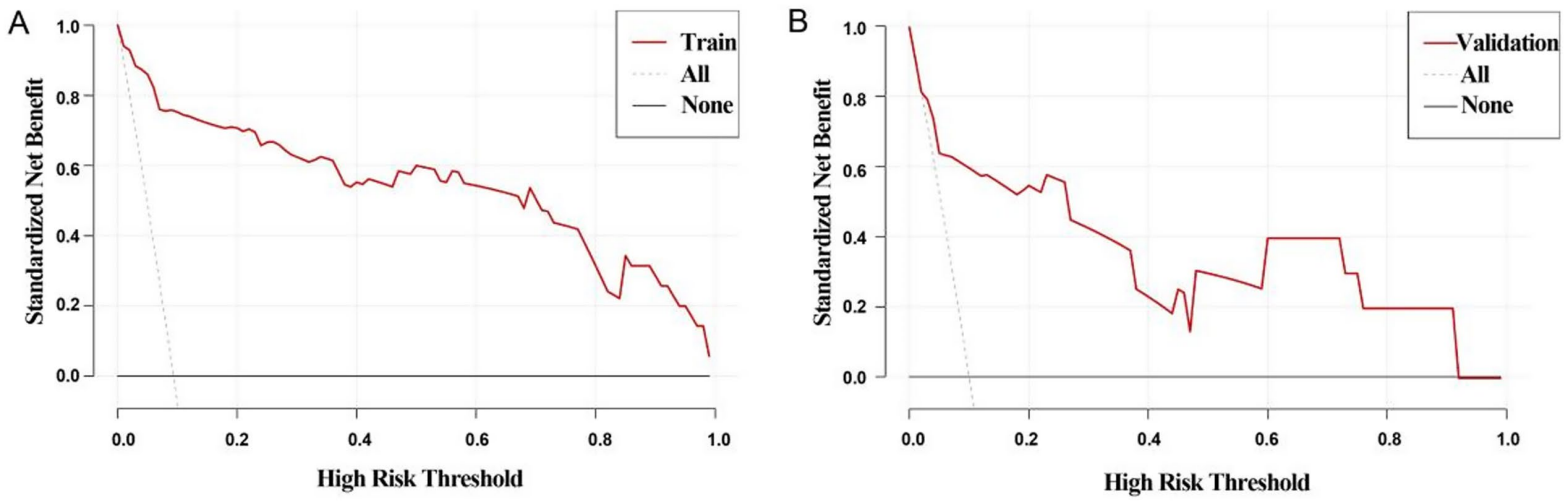
Decision curve analysis curves for training group **(A)** and validation group **(B)**, All line: indicates that all patients had complications; None line: indicates that none of the patients had complications. If the Train line was above both the All and None lines, indicating that the model performed better in the probability range.

External validation was conducted to evaluate the predictive performance of the model. The AUC of the ROC curve in the training cohort was 0.964 (95% CI: 0.937–0.991), and in the validation cohort was 0.846 (95% CI: 0.664–1.000), indicating excellent discrimination (Figures 3A,B). The calibration curve demonstrated strong agreement between predicted and actual probabilities, with predicted values closely aligning with the 45 ° reference line across all quantiles (Figures 4A,B). DCA curve showed that the model provided greater net benefit than the “treat none” strategy at all thresholds and outperformed the “treat all” strategy when the threshold exceeded 0.1. Notably, in the validation cohort, net benefit decreased by approximately 15% in the 0.2–0.4 range, possibly reflecting slight calibration drift. However, model performance remained stable at thresholds > 0.4 (Figures 5A,B). Overall, this nomogram offers reliable clinical guidance in predicting complications after UBE for TSS, particularly for clinical decision-making scenarios with a risk threshold > 0.4.

## Discussion

Surgical treatment of TSS remains a significant challenge in spinal surgery, with the core focus on achieving effective decompression while minimizing the risk of perioperative complications. Recently, UBE has emerged as a minimally invasive option for TSS due to its advantages of enhanced visualization and flexible manipulation. However, given the complex anatomical structure of the thoracic spine and the low tolerance of the spinal cord to surgical stress, systematic analysis of postoperative complications after UBE is still limited. Identifying high-risk factors and establishing a risk warning model are crucial for clinical practice. In this retrospective study of 375 patients who underwent UBE for TSS, we developed a nomogram for predicting postoperative complications for the first time. The results demonstrated that diabetes, SC/ECA ≥ 55%, and intramedullary high signal were markedly associated with poor outcomes. The model showed good discrimination in both the training and validation cohorts, providing an important tool for preoperative risk assessment, patient stratification, and individualized treatment planning.

Although UBE has markedly reduced the incidence of postoperative complications in TSS (8–10% in our study vs. up to 25.2% in traditional open surgery), CSF leakage and neurological deterioration remain concerning, especially in high-risk patients with complex anatomy. This highlights that while minimally invasive techniques reduce surgical trauma, they cannot fully overcome the inherent technical challenges of thoracic spine surgery, such as dense adhesions between the dura and ligaments and limited spinal cord buffering space. Therefore, accurate preoperative identification of high-risk patients, optimization of surgical strategies, and enhanced postoperative monitoring are essential to further ensure surgical safety, which underscores the clinical value of this study.

Regarding patient-related baseline factors, diabetes and low preoperative JOA scores were identified as independent risk factors for postoperative complications. Diabetes was associated with a four-fold increased risk of complications, the reason maybe that chronic hyperglycemia promotes the accumulation of advanced glycation end-products, leading to microvascular basement membrane thickening and endothelial dysfunction, which compromise tissue perfusion and oxygen delivery, impairing wound and neural tissue healing (13). Moreover, diabetic patients exhibit impaired neutrophil chemotaxis and macrophage polarization imbalance, substantially increasing the risk of postoperative infection (14). In our study, all six patients with poor wound healing had pre-existing type 2 diabetes. The inverse association between JOA scores and complications (36.4% risk reduction per point increase) reflects the consequence of severe baseline neurological impairment. A low preoperative JOA score, reflecting severe neurological impairment, suggests irreversible changes such as axonal degeneration, demyelination, and neuronal apoptosis due to prolonged spinal cord compression (15). These conditions reduce the spinal cord’s tolerance to mechanical stress during surgery. Furthermore, the thoracic spinal cord’s unique vascular anatomy makes it a watershed region with fewer anastomoses. Chronic compression-induced microcirculatory dysfunction may predispose the tissue to ischemic preconditioning, rendering it vulnerable to reperfusion injury after decompression (16). These findings are consistent with those reported by Hitchon et al. (17) and emphasize the importance of comprehensive preoperative assessment and optimized perioperative management, particularly in patients with diabetes or severe neurological deficits.

In terms of preoperative imaging parameters, SC/ECA ≥ 55%, intramedullary high signal, and TK angle ≥ 45 ° were identified as significant risk factors for postoperative complications. SC/ECA, calculated from axial MRI, provides a precise quantification of spinal canal stenosis. When this ratio exceeds 55%, the spinal cord’s buffering capacity is drastically reduced, often accompanied by epidural fat obliteration and altered CSF dynamics, which increase the risk of iatrogenic injury during surgery. Intramedullary high signal is widely considered a marker of spinal cord damage and is associated with poor neurological recovery (18). These hyperintense regions exhibit increased vulnerability to secondary injury due to edema and inflammation, while underlying microvascular impairment and metabolic abnormalities further hinder postoperative recovery (19). In our study, 5 of 8 patients with postoperative neurological deterioration had preoperative intramedullary high signal. TK angle ≥ 45 ° also warrants attention, as such deformity causes the spinal cord to shift anteriorly and adhere to the posterior vertebral body, reducing the dural sac’s buffering space by 30–40% (20). Moreover, the abnormal stress distribution associated with kyphosis can form dural folds within the decompression zone, creating “blind spots” that increase the risk of instrument-induced injury. Hu (21) et al. stated that a kyphosis angle ≥ 45 ° increases the complication rate of decompression surgery by 1–3.5-fold, which aligns with our clinical observations. These findings underscore the necessity of thorough preoperative evaluation of imaging features and the formulation of individualized surgical strategies to optimize procedural safety.

From a surgical perspective, multisegment lesions were identified as the most potent independent risk factor for complications following UBE. These cases require more extensive decompression, increasing dural exposure and surgical duration (22). Moreover, continuous multilevel decompression can lead to cumulative spinal cord traction, especially in the presence of dural adhesions, which act as anchor points that amplify mechanical stress and exacerbate neurological injury risk (23). The resulting nonlinear risk escalation likely contributes to the elevated OR. However, the wide confidence intervals observed may reflect limitations in statistical power due to the relatively small sample size of the multisegment subgroup and low event rates of complications. Multilevel decompression may also compromise spinal stability, particularly in patients with significant TK. In our cohort, 36% of patients with TK angle > 45 ° and multisegment lesions developed postoperative complications, predominantly neurological deterioration. Dural adhesions obscure normal anatomical planes, making dural separation from ligamentous tissue difficult, thereby increasing the risk of CSF leakage (notably, 53% of CSF leak cases in our study involved dural adhesions) and complicating hemostasis. Importantly, dural adhesions and multisegment lesions had a synergistic effect, increasing the risk of CSF leakage by 7.2-fold when both were present. This finding suggests that for such high-risk patients, modified strategies such as “floating decompression” or staged surgeries should be considered.

Regarding perioperative indicators, prolonged hospitalization was associated with complications through multiple pathways. This may reflect the cumulative effects of surgical trauma, especially in multisegment surgery or cases involving dural adhesions, where increased operative time and tissue injury delay recovery (24). Alternatively, prolonged hospitalization may serve as an early warning sign of impending complications (25). Among patients hospitalized for more than 10 days, 38.5% subsequently developed infections or neurological deterioration. This association was more pronounced in patients with diabetes or TK, indicating a more complex clinical course and highlighting the need for early multidisciplinary intervention and close postoperative monitoring.

This study successfully developed and validated a nomogram integrating multidimensional perioperative risk factors for predicting complications following UBE in TSS patients. Multisegment lesions (25 points) and SC/ECA ≥ 55% (20 points) were identified as the most predictive factors, aligning with the anatomical and surgical challenges discussed earlier. The inclusion of diabetes (10 points) and dural adhesions (10 points) further validated the synergistic effects of systemic metabolic and local pathological changes on complication risk. The model demonstrated excellent predictive performance, with AUCs of 0.964 and 0.846 in the training and validation cohorts, respectively, markedly outperforming existing predictive tools for spinal surgery complications [e.g., AUC 0.782–0.818 for surgical site infection models after lumbar discectomy (26); AUC 0.772–0.792 for deep vein thrombosis models following posterior lumbar fusion (27); AUC 0.722–0.743 for infection models after degenerative lumbar instrumentation (28)]. Calibration curves indicated high concordance between predicted and observed probabilities, while DCA curve analysis confirmed a clear clinical net benefit when the threshold probability exceeded 0.1. A key strength of this model is its ability to transform complex multifactorial interactions into an intuitive point-line system, enabling rapid preoperative risk assessment and providing an evidence-based foundation for personalized surgical planning, thereby optimizing both “decompression precision” and “complication control.”

Nevertheless, several limitations should be acknowledged. Firstly, although external validation was performed, the validation cohort was relatively small (*n* = 100) and sourced from a single center, which may limit the model’s generalizability to other institutions. Secondly, although the model integrates nine key predictive indicators, it does not encompass all potential influencing factors—particularly operator experience and surgical instrument type, which are important technical variables. Thirdly, due to the limited sample size, we were unable to develop complication-specific models (CSF leakage or neurological deterioration), despite potential differences in their risk profiles. These limitations suggest that future research should aim to improve model performance through multicenter, prospective studies, incorporating more comprehensive predictors and long-term follow-up data to further enhance the clinical utility of this tool.

## Conclusion

This study successfully developed and validated the first predictive model for postoperative complications following UBE in TSS patients. The model incorporates multiple key factors, including diabetes, preoperative JOA score, SC/ECA ratio, intramedullary high signal, TK angle, intraoperative blood loss, dural adhesions, number of multisegment lesions, and hospital stays, which were shown to collectively influence complication risk. The model demonstrated high predictive accuracy and strong clinical applicability. It provides a valuable tool for preoperative risk assessment and individualized treatment planning, contributing to the precision and safety of minimally invasive thoracic spine surgery and ultimately improving patient outcomes.

## Data Availability

The raw data supporting the conclusions of this article will be made available by the authors, without undue reservation.
